# Third trimester fetal 4D flow MRI with motion correction

**DOI:** 10.1002/mrm.30411

**Published:** 2025-01-09

**Authors:** Reagan M. Tompkins, Takashi Fujiwara, Eric M. Schrauben, Lorna P. Browne, Joost van Schuppen, Sally‐Ann Clur, Richard M. Friesen, Erin K. Englund, Alex J. Barker, Pim van Ooij

**Affiliations:** ^1^ Department of Radiology & Nuclear Medicine, Amsterdam University Medical Center University of Amsterdam Amsterdam The Netherlands; ^2^ Department of Radiology, Section of Pediatric Radiology, Children's Hospital Colorado University of Colorado Anschutz Medical Campus Aurora Colorado USA; ^3^ Department of Pediatric Cardiology, Emma Children's Hospital, Amsterdam University Medical Center University of Amsterdam Amsterdam The Netherlands; ^4^ Department of Pediatrics, Section of Cardiology, Children's Hospital Colorado University of Colorado Anschutz Medical Campus Aurora Colorado USA; ^5^ Department of Bioengineering University of Colorado Anschutz Medical Campus Aurora Colorado USA; ^6^ Amsterdam Cardiovascular Sciences, Amsterdam University Medical Center University of Amsterdam Amsterdam The Netherlands

**Keywords:** 4D flow, fetal CMR, motion correction

## Abstract

**Purpose:**

To correct maternal breathing and fetal bulk motion during fetal 4D flow MRI.

**Methods:**

A Doppler‐ultrasound fetal cardiac‐gated free‐running 4D flow acquisition was corrected post hoc for maternal respiratory and fetal bulk motion in separate automated steps, with optional manual intervention to assess and limit fetal motion artifacts. Compressed‐sensing reconstruction with a data outlier rejection algorithm was adapted from previous work. Pre‐ and post–motion correction comparison included qualitative visibility of vasculature on phase‐contrast MR angiograms (five‐point Likert scale), conservation of mass of the aortic isthmus, ductus arteriosus, and descending aorta, and coefficient of variation of flow along the descending aorta.

**Results:**

Twenty‐nine third trimester acquisitions were performed for 15 healthy fetuses and two patients with postnatally confirmed aortic coarctation during a single examination for each participant. Only 15/27 (56%) of all volunteers and 1/2 (50%) of all patient precorrection acquisitions were suitable for flow analysis. Motion correction recovered eight “failed” acquisitions, including one patient, with 24/29 (83%) suitable for flow analysis. In the 15 viable uncorrected volunteer acquisitions, motion correction improved phase‐contrast MR angiograms visibility significantly in the ductus arteriosus (from 4.0 to 4.3, *p* = 0.04) and aortic arch (3.7 to 4.0, *p* = 0.03). Motion correction improved conservation of mass to a median (interquartile range) percent difference of 5% (9%) from 14% (24%) with improvement shown in 14/15 acquisitions (*p* = 0.002), whereas coefficient of variation changes were not significantly different (uncorrected: 0.15 (0.09), corrected: 0.11 (0.09), *p* = 0.3).

**Conclusions:**

Motion correction compensated for maternal and fetal motion in fetal 4D flow MRI data, improving image quality and conservation of mass.

## 1 INTRODUCTION

Accurate assessment of the fetal circulation plays a vital role in confirming late gestation diagnoses, planning for delivery, and preparing for surgical interventions.^1^, ^2^, ^3^, ^4^ Whereas echocardiography is the clinical standard for the evaluation of congenital heart defects (CHD), it is increasingly challenging at later gestational ages due to ossification of the fetal skeleton, decreasing amniotic fluid, and suboptimal fetal position. These issues lead to difficult visualization of the great vessels and inconclusive CHD diagnoses.^5^ In high‐risk cases, fetal cardiovascular MRI (CMR) has become adjunct to echocardiography for informing CHD diagnoses in the third trimester.^6^, ^7^, ^8^, ^9^ Current fetal CMR protocols primarily focus on anatomical scans, such as black blood imaging^10^, ^11^; however, visualization of blood flow in the fetus can provide crucial insight into abnormal fetal vasculature. Therefore, robust and comprehensive functional 3D flow assessments can provide valuable insight into a range of CHDs, including coarctation of the aorta (CoA) and Tetralogy of Fallot, thus enhancing the diagnostic and prognostic capabilities of fetal CMR.^12^, ^13^

Motion corruption is a major inherent obstacle for fetal CMR and is particularly detrimental for longer acquisitions such as those measuring volumetric (3D) cardiac time‐resolved blood flow. Artifacts related to motion, such as ghosting or blurring, frequently arise from a combination of maternal respiration, spurious fetal motion, and fetal cardiac motion. Regarding the latter, recent studies have addressed the initial barrier to dynamic time‐resolved fetal cardiac imaging caused by the lack of access to a fetal electrocardiogram signal, which is normally used to bin acquired data according to periodic heart motion. These innovations include retrospective metric optimized gating,^14^ cardiac self‐gating,^15^ or Doppler ultrasound (DUS) gating.^16^ Despite these advancements, bulk motion artifacts persist because unavoidable fetal stochastic movements, such as rotation and displacement, occur in all directions during an acquisition. To limit the impact of such motion, highly accelerated data acquisition strategies have been employed to reduce scan duration and therefore the likelihood of fetal bulk motion.^17^

2D phase‐contrast MRI for blood flow quantification through a single plane has been used to evaluate fetal blood flow with MRI.^8^, ^18^ To capitalize on the available signal from 2D acquisitions and provide volumetric flow measurements, studies have explored slice‐to‐volume reconstructions using highly accelerated 2D phase‐contrast MRI radial acquisitions^19^ or velocity values derived from single‐shot balanced steady‐state free precession imaging.^20^ These approaches, however, require multiple overlapping acquisitions and robust registration techniques to reduce the effects of fetal motion between slice acquisitions, leading to long scan durations. Additionally, these techniques rely on estimations of fetal heart rates from the acquired data, rather than direct measurements, and are vulnerable to through‐slice motion.

4D flow MRI provides 3D velocity encoding over cardiac time for a given volume and has established itself as a tool to evaluate pediatric and adult cardiac hemodynamics.^21^ Whereas fetal 4D flow MRI holds the potential to offer comparable quantitative measurements with the added clinical advantage of volumetric flow visualization, it faces significant challenges. Achieving sufficient SNR in 4D flow MRI is hindered by limitations in temporal and spatial resolution for fetal‐scale measurements and constrained scan acquisition times, due to the risk of stochastic fetal motion. Although 4D flow MRI has been successfully demonstrated in fetal imaging, these studies have not been conducted with the incorporation of motion‐correction techniques^13^, ^22^ and there remains a need for further advancements in this area.

Here, we demonstrate a method for retrospective motion correction of fetal 4D flow data, accounting for motion related to maternal respiration and fetal bulk motion. The basis for this approach is an undersampled Cartesian pseudo‐spiral data acquisition with Doppler ultrasound gating, maternal respiratory binning, rigid image registration for fetal motion, and compressed sensing (CS) reconstruction. We hypothesize that this acquisition and motion‐correction strategy will provide better image quality and more reliable flow quantification than uncorrected data.

## 2 METHODS

### 2.1 Participant inclusion and setup

This study (COMIRB 18‐2154) was approved by the University of Colorado, Anschutz Medical Campus Institutional Review Board and Children's Hospital Colorado (Aurora, Colorado). Prospective, informed written consent was obtained from all pregnant women prior to participation in this study. Women with healthy singleton fetuses in their third trimester of pregnancy were included as volunteers. Two women were included that had concern on clinical echocardiography for CoA, a condition characterized by a narrowing in the aortic isthmus, with or without a hypoplastic aortic arch.^23^

All imaging was performed on a 3 T MRI scanner (Ingenia or Elition X; Philips Healthcare, Best, The Netherlands) at Children's Hospital Colorado. Participants were placed in the left lateral decubitus position or supine according to patient comfort, and total fetal CMR exam time was limited to 60 min. A respiratory belt was utilized to record maternal respiratory motion throughout scanning, and the exam was performed under free breathing conditions. An MR‐compatible DUS transducer (smart‐sync, Northh Medical GmbH, Hamburg) was fixed via elastic belt on the mother's abdomen over the fetal heart to provide fetal cardiac gating.^16^ The DUS transducer was positioned over the fetal heart using a handheld display and headphones that visually and sonically depicted the Doppler signal of the fetal heart motion. Once the transducer position produced a steady signal of the heart motion, a 32‐channel torso coil was placed anteriorly, with care taken not to disturb the DUS transducer.

### 2.2 4D flow acquisition

**FIGURE 1:**
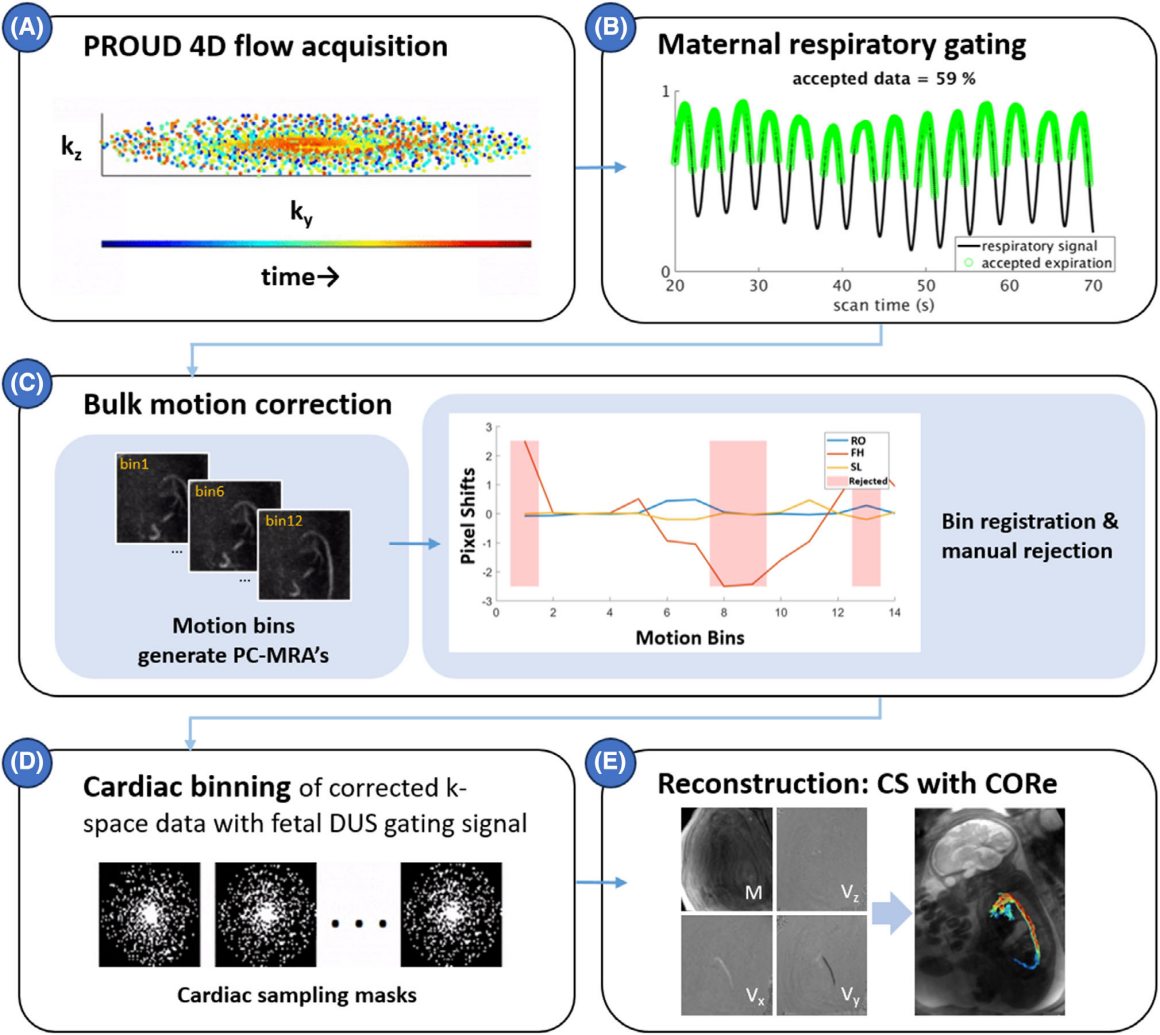
(A) PROUD data is continuously sampled with a pseudo‐spiral k‐space trajectory. (B) Data is retrospectively limited to the maternal expiratory phase, roughly 60% of data. (C) For bulk motion detection, data is binned into dynamic bins with a temporal resolution of 15 s. CS reconstruction and PC angiogram calculation are performed for all bins. Then, rigid monomodal registration of the angiogram frames corrects for fetal bulk motion over the course of data acquisition. The red blocks denote rejected bins, either for large translational shifts or for a loss of signal observed in the bin's angiogram. The obtained translations of the remaining bins were directly applied to the original k‐space data. (D) A Doppler ultrasound gating device provides temporal fetal cardiac information that guides reconstruction. (E) 4D flow is reconstructed though CS with CORe. 4D flow is depicted by the magnitude and three orthogonal velocity (*V*_x_, *V*_y_, *V*_z_) images at peak systole. An animated version of this figure is found in Video S1. CORe, compressive recovery with outlier rejection; CS, compressed sensing; *M*, magnitude; PROUD, prospective undersampling in multiple dimensions.

The fetal MR protocol included initial localizers to identify the position of the fetus and to verify the placement of the DUS transducer over the fetal heart.^24^ For the 4D flow sequence, an overview of the acquisition and motion‐correction pipeline is shown in Figure 1 (animation shown in Video S1). Briefly, CS‐accelerated 4D flow data were acquired in a pseudo‐spiral Cartesian trajectory (Figure 1A), with prospective undersampling in multiple dimensions (PROUD), as reported previously for aortic 4D flow MRI.^25^ The free‐running PROUD acquisition uses a predetermined trajectory list, calculated for the intended matrix size and imported to the scanner prior to the exam.^25^ During acquisition, DUS gating provided cardiac triggering times to the scanner that informed retrospective cardiac gating, with incoherent k‐space undersampling per cardiac frame. When scan time allowed, multiple PROUD 4D flow sequences were performed per subject during a single examination to vary acquired slice resolution and potentially increase the chance of a usable image. The sequence employed the following acquisition parameters^22^, ^26^: parasagittal 3D volume covering all fetal cardiac structures, acquired spatial resolution = 2.5 × 2.5 × 1.25 mm^3^ or 2.5 mm^3^ isotropic, reconstructed to 1.25 mm isotropic and 38–50 ms temporal resolution (10 cardiac frames irrespective of fetal heart rate), velocity encoding = 150 cm/s with four‐point Hadamard for improved velocity‐to‐noise ratio,^27^ FOV = 300 × 300 mm^2^, TR/TE/flip angle = 2.64–3.73 ms/1.65–2.21 ms/6.5°, target acceleration R = 2.5–5, and scan time = 92–441 s.

### 2.3 4D flow reconstruction and motion correction

All image reconstruction was performed offline. Reconstruction of the 4D flow k‐space data was completed in MatLab R2021a (MathWorks, Natick MA) with the Berkeley Automated Reconstruction Toolbox (BART)^28^ and ReconFrame (Gyrotools, Zurich, Switzerland) at Amsterdam University Medical Center (UMC) (Amsterdam, The Netherlands). Postprocessing steps included correction for maternal respiratory and fetal bulk motion in separate steps, with optional manual intervention to assess and limit the presence of extreme fetal motion for each acquisition, and finally, gated CS reconstruction with a data outlier (wrongly binned readouts or readouts corrupted by motion) rejection algorithm adapted from previous work.^29^ Total reconstruction required approximately 45 min. Details regarding each step are outlined below.

#### 2.3.1 Respiratory gating

The maternal respiratory signal was obtained from the respiratory belt signal when available. Gating efficiency was set to accept roughly 60% of the data nearest to the expiration plateau. This generalized acceptance window follows from previous studies using prospective navigator gating in 4D flow MRI to optimize scan efficiency and image quality.^30^, ^31^

#### 2.3.2 Bulk motion correction

3D bulk motion correction was achieved by sorting the raw k‐space data into consecutive chronological bins at a temporal resolution of 15 s/bin. This temporal length was chosen to be long enough to effectively average residual maternal breathing motion while also short enough to capture varied fetal bulk motion states over several minutes of scanning. Bins were reconstructed with CS using temporal total variation over the bin dimension and, with time‐averaged magnitude and velocity information, a phase‐contrast MR angiogram (PC‐MRA) was generated for each dynamic bin to represent each bulk motion phase. Image registration between angiographic bins was performed using the MatLab (MathWorks) Image Processing Toolbox, R2021a. First, each PC‐MRA was 4× interpolated using cubic interpolation to improve registration results. An affine translation‐only registration (maximum iterations = 100, gradient tolerance = 1e‐4) was then determined between angiographic bins, with a manually selected bin, based on visually assessed maximum available signal in fetal anatomy, serving as reference. Rigid translations were stored for all three orthogonal directions for each bulk motion bin. Based on this estimated motion, bins with extreme motion, corresponding to a large translation or bins in which the targeted fetal anatomy was not visible, were manually excluded from the scan data. The 3D translations for the remaining bulk motion bins were then directly applied to the original k‐space data.

#### 2.3.3 Reconstruction with CORe

The DUS signal enabled retrospective cardiac gating, and 10 cardiac bins were generated. PROUD reconstruction with CS^25^ was adapted to incorporate a publicly available algorithm for additional motion suppression, compressive recovery with outlier rejection (CORe).^29^ The CORe reconstruction method assumes that physiological motion, which occurs at time scales longer than a readout, induces additive outliers on the readout level in k‐space rather than simply noise at individual k‐space samples. The model imposes group sparsity on these outlier readouts, which would otherwise result in imperfect k‐space data binning, and therefore reduces image artifacts. CORe's CS approach includes an additional optimization parameter $\lambda_{2}$ to control the extent of outlier rejection at the readout level. This is shown in Equation (1),

(1) $$\hat{x}=\arg\min\left\{\frac{1}{\sigma^{2}}{\left\|\mathrm{Ax}-\left(y-v\right)\right\|}_{2}^{2}+\lambda_{1}{\left\|\mathrm{Wx}\right\|}_{1}+\lambda_{2}{\left\|v\right\|}_{2,1}\right\},$$

$\hat{x}$ denotes the reconstructed CMR image, x the underlying image, y the measured k‐space data, v the estimated outliers in data, $\sigma^{2}$ the variance of additive white Gaussian noise, A the sensing matrix, and W the 4D undecimated wavelet transform in the spatiotemporal domain, acting over 3D space and cardiac time.^32^ Tuning parameters were determined using multiple reconstructions in a single fetal 4D flow data set, with raw k‐space scaled to 10% of its maximum absolute value. This tuning, coarse and fine, was optimized for a set of target values: minimized coefficient of variation of net flow through a preestablished segmentation of the descending aorta (DAo), maximized PC‐MRA SNR and peak flow to limit the potential effects of smoothing. The resulting parameter values were used for all subsequent reconstructions (*λ*_1_ = 2.55 × 10^−4^ and *λ*_2_ = 1.125 × 10^−1^).

#### 2.3.4 Uncorrected data

For comparison, a separate group of “uncorrected” images was reconstructed from the PROUD 4D flow acquisitions with CS reconstruction with a temporal total variation regularization (λ=0.01) and no additional motion‐correction steps or data removal.

### 2.4 Data analysis

Vessel segmentation was performed in 3D Slicer (version 5.2.2)^33^ and included the ductus arteriosus (DA), main pulmonary artery, ascending aorta, aortic arch, carotid arteries (if visible), and the DAo to the point of iliac bifurcation. If segmentation of the DAo and ductal insertion were deemed incomplete in both reconstructions, the acquisition was excluded from further analysis and reported as “failed.” Failed uncorrected reconstructions, which had corresponding motion‐corrected reconstructions with successful segmentations, were labeled as “recovered.”

#### 2.4.1 Qualitative metrics

The uncorrected and motion‐corrected datasets with sufficient image quality in both uncorrected and motion‐corrected reconstructions were assessed by three clinical expert observers (one pediatric cardiologist, r.m.f., with 7 years of experience with pediatric cardiac MRI; two pediatric radiologists, j. vs. and l.p.b., with 11 and 18 years of experience with pediatric cardiac MRI) in a blinded, randomized evaluation of the ability to visualize the relevant fetal vasculature in each PC‐MRA volume on a five‐point Likert scale (with 0 representing a feature not present). Scored features included the DAo, aortic arch, DA, and umbilical veins.

#### 2.4.2 Quantitative metrics

**FIGURE 2:**
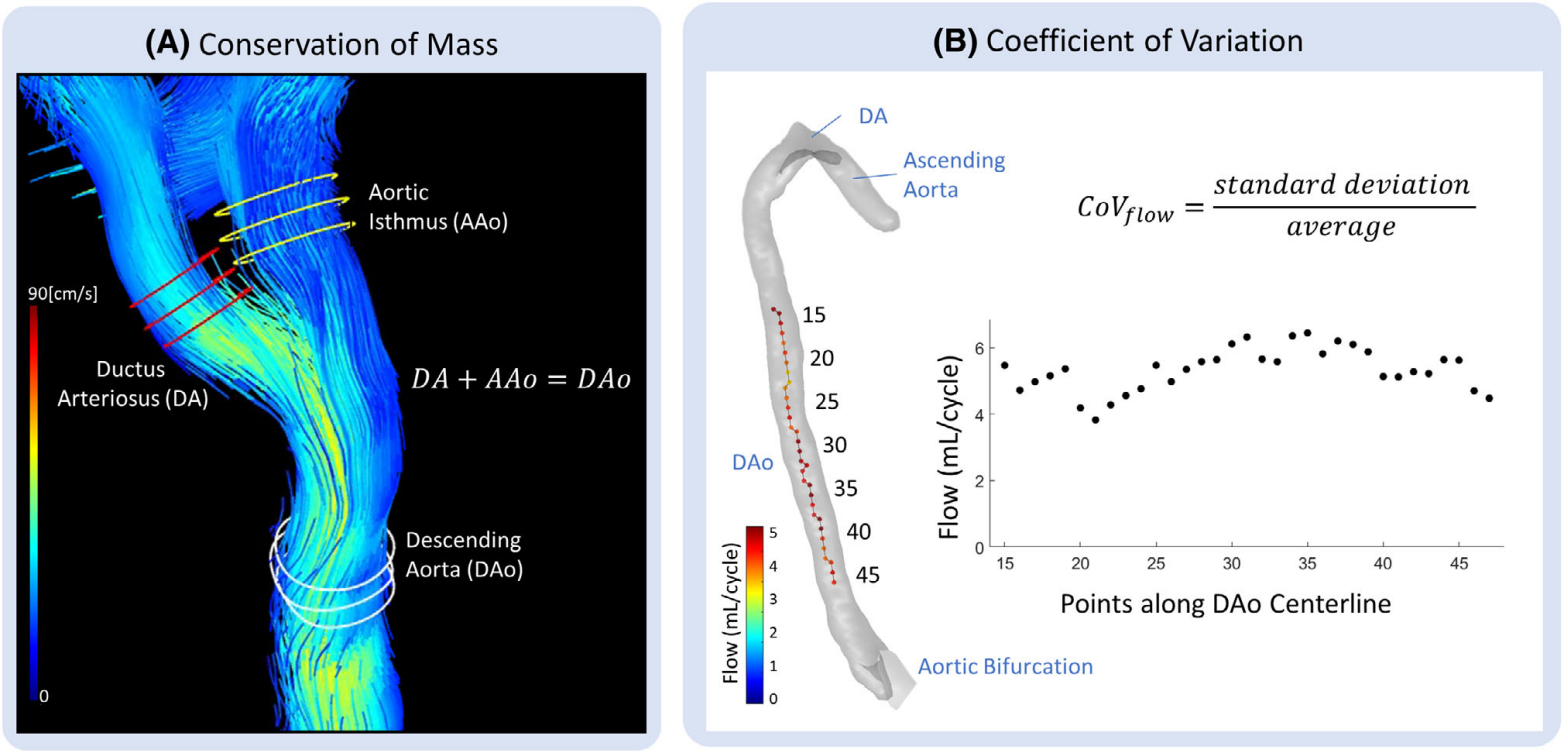
Internal validation metrics for the evaluation of the motion‐correction performance. (A) CoM was evaluated for net flow at the ductal insertion into the aorta, with three equidistant planes placed at each vessel and time‐resolved contours drawn for each plane. (B) CoV was evaluated for net flow using automated contours on a static segmentation along the descending aorta. CoM, conservation of mass; CoV, coefficient of variation.

4D Flow was internally validated using conservation of mass (CoM) and the coefficient of variation (CoV), demonstrated in Figure 2. Flow within the major fetal vessels was visualized in GTFlow (Gyrotools, Zurich, Switzerland) for both motion‐corrected and uncorrected images. The principle of CoM states that in an absence of control volume source or sinks, an incompressible fluid (such as blood) should have equal flux (or net flow volume) into and out of the control volume, and the concept serves as a within subject validation measure.^34^ Thus, three 20 × 20 mm planes, spaced 1.25 mm apart, were semiautomatically placed orthogonally to each of the three vessels (DA, aortic isthmus, and DAo) near the insertion of the DA into the aorta. At each plane, a time‐resolved contour was manually drawn, based on the velocity images and a derived velocity magnitude overlay, and the net flow through the contour was determined and averaged over the three planes. CoM was evaluated for flow divergence through the ductal insertion, that is, DA+aortic isthmus=DAo, and expressed as a percent difference in flow values for the left‐ and right‐hand side of the equation.

Furthermore, the CoV for net flow through the DAo was used to measure flow consistency along the DAo, using the publicly available Amsterdam UMC Flow Processing app in MatLab R2021a (MathWorks) for PROUD 4D flow data (https://github.com/schrau24/FlowProcessing). Based on a provided segmentation volume, a centerline was automatically computed along the DAo, and equally spaced, static contours based on the segmentation extent were drawn at one voxel spacing along the centerline. Net flow rate was computed at each contour along a section of the DAo for each of the 10 cardiac phases, extending from a point on the DAo corresponding to the level of the proximal aortic root to a point just proximal to aortic bifurcation.

#### 2.4.3 Statistical analysis

Interobserver agreement of image quality and feature definition were assessed using the average intraclass coefficient (ICC) with a two‐way model for consistency. The feature scores were averaged over observers, and reported *p*‐values were determined using the Wilcoxon signed‐rank test. Differences in CoM and CoV between uncorrected and motion‐corrected datasets were compared using a Wilcoxon signed‐rank test; *p* < 0.05 was considered statistically significant.

## 3 RESULTS

### 3.1 Application of motion correction

**FIGURE 3:**
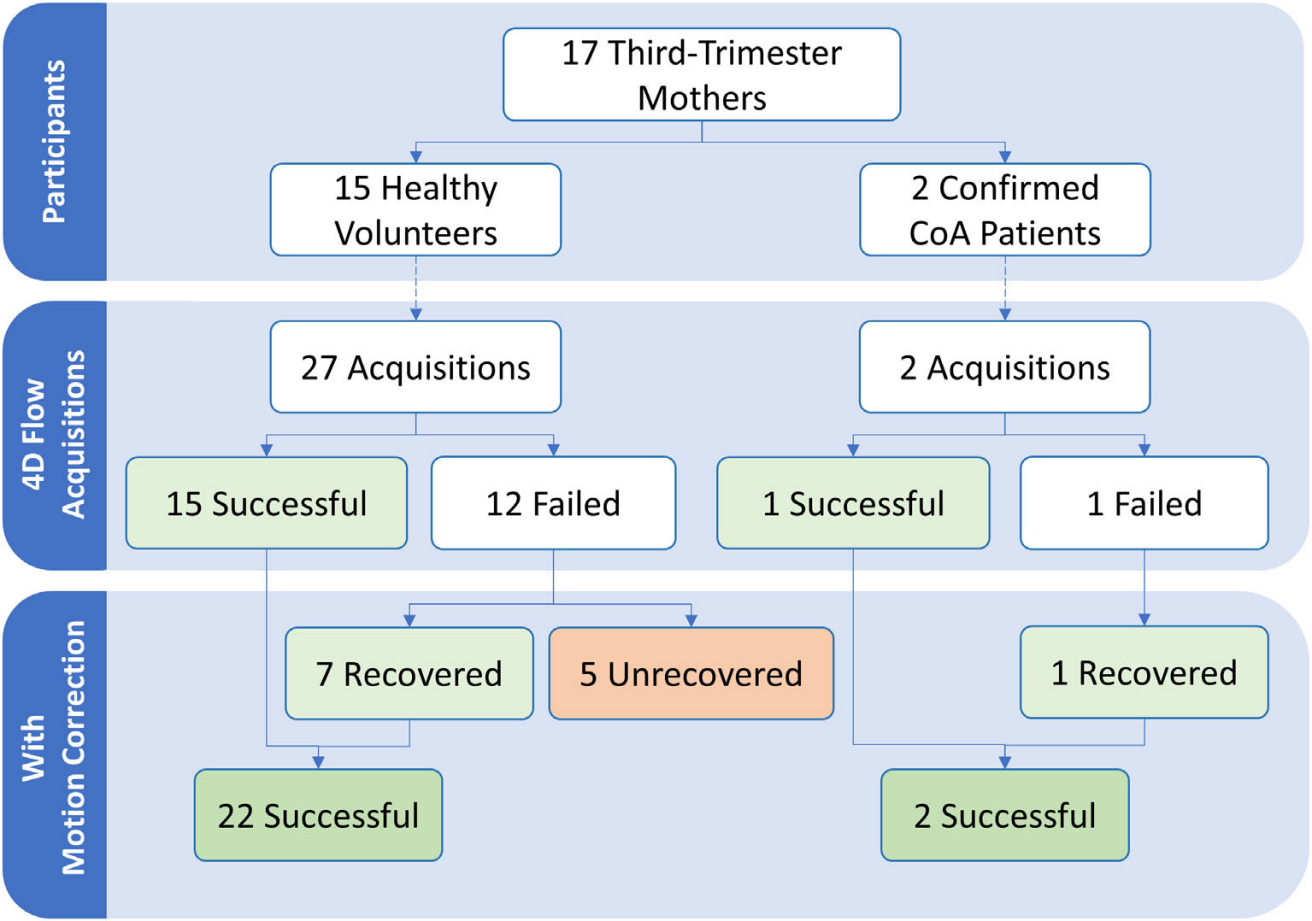
Summary of participant groups and acquisitions. Successful acquisitions had sufficient image quality prior to motion correction and were included in quantitative analysis. Failed acquisitions were deemed as “recovered” by motion correction if the image quality had improved to allow segmentation and flow analysis.

A total of 15 healthy volunteers were recruited (mean maternal age = 34.7 years, mean gestational age = 34.1 weeks, gestational age range = 32–37 weeks). In addition, two patients, who were confirmed as CoA postnatally (maternal age = 31 years, 26 years; gestational age = 32 weeks, 30.5 weeks), were included to evaluate the motion‐correction pipeline's performance in the target clinical population. Across all 17 participating mothers, a total of 29 PROUD acquisitions were obtained with a mean scan time of 228 ± 85 s. A summary of the participant groups and the results of each acquisition are given in Figure 3. Of the 29 4D flow scans, 13 were labeled as “failed” uncorrected acquisitions, with too sparse of signal in the target vessels to be segmented. Motion correction successfully recovered eight of the failed acquisitions, including one patient with no viable uncorrected images. However, five scans could not be recovered and remained as failed acquisitions due to continuous bulk motion throughout scanning as observed in the binned bulk motion reconstruction, leading to angiographic signal dropout within bins and failure in registration. In three of the 15 mothers with healthy fetuses, 4D flow was entirely unsuccessful (*n* = 4 scans; in two of these mothers, only one acquisition was attempted). In Table S1, all acquisitions are described in terms of individual scan details and usability (or recoverability) of the uncorrected images.

Quantitative comparison was performed for the 15 healthy fetal acquisitions in which the uncorrected reconstructions had sufficient image quality for full analysis prior to motion correction. Motion correction performance was assessed through observer scoring and 4D flow quantification. The eight aforementioned “recovered” scans were excluded from quantitative comparison because the baseline uncorrected segmentation was unavailable. Within the two CoA patients, motion correction successfully recovered one failed patient acquisition; a quantitative comparison of pre‐ and post‐motion correction was completed for the other.

**FIGURE 4:**
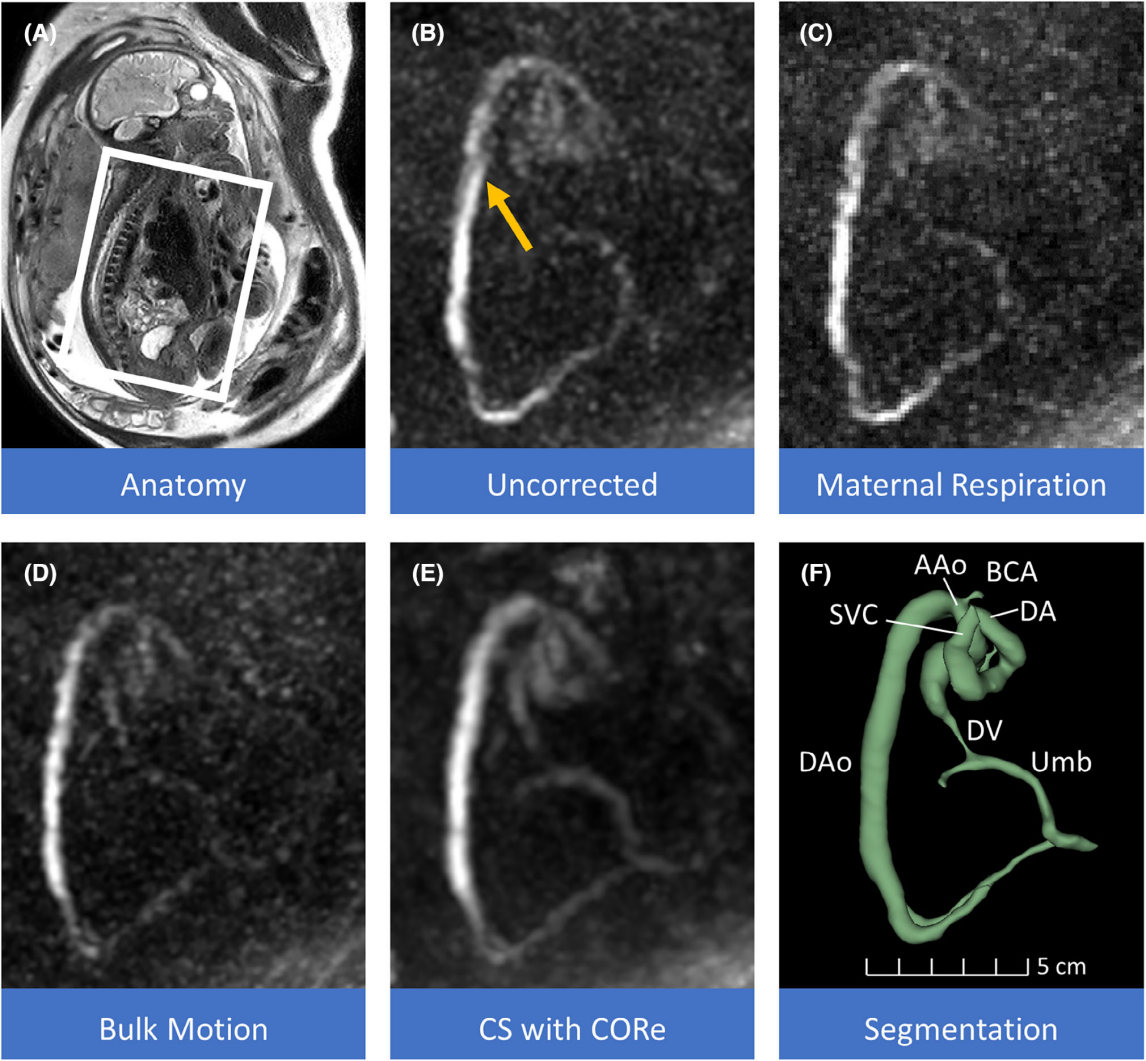
Example step‐by‐step motion correction in 4D flow. (A) The ROI is denoted by the white box in the sagittal anatomical view. (B–E) 4D flow is represented by maximum intensity projections of PC‐MRA covering the fetal cardiac region, (B) with the original uncorrected PC‐MRA. The yellow arrow indicates blurring along the DAo from motion. (C) In maternal respiratory gating, 40% of the data is removed to compensate for inspiratory motion. (D) The blurring from motion in the DAo (yellow arrow in panel B) is corrected with bulk motion, in which a further 25% of data was rejected in this particular acquisition. (E) CS with CORe provides noise reduction in the final reconstruction with motion correction with greater visualization of vasculature. (F) The value of motion correction in 4D flow analysis is exemplified by the corresponding segmentation of (E). Data for the final reconstruction comprised of 45% of the original acquired data in this example. This acquisition is reported as Acq. 9 in Figure 4. AAo, ascending aorta; BCA, brachiocephalic artery; CORe, compressive recovery with outlier rejection; CS, compressed sensing; DA, ductus arteriosus; DAo, descending aorta; DV, ductus venosus; PC‐MRA, phase‐contrast MR angiogram; ROI, region of interest; SVC, superior vena cava; Umb, umbilical vessels.

Figure 4 demonstrates a stepwise example of the motion‐correction method's performance with the PC‐MRA of the major fetal vessels of one participant showing uncorrected image with a disruption in the descending aorta indicated by the yellow arrow, maternal respiration correction, then with bulk motion correction added, and finally with CS with CORe reconstruction added. Respiratory gating in Figure 4C reduces some blurring of the descending aorta. The application of image registration, Figure 4D, with fetal bulk motion correction further reduces blurring of the aorta observed in the original image. The final CS with CORe demonstrates the capability of the outlier rejection method to reduce noise and recover more detail in the umbilical and more peripheral vessels (Figure 4E), thus enabling the segmentation of major fetal cardiovascular features (Figure 4F).

**FIGURE 5:**
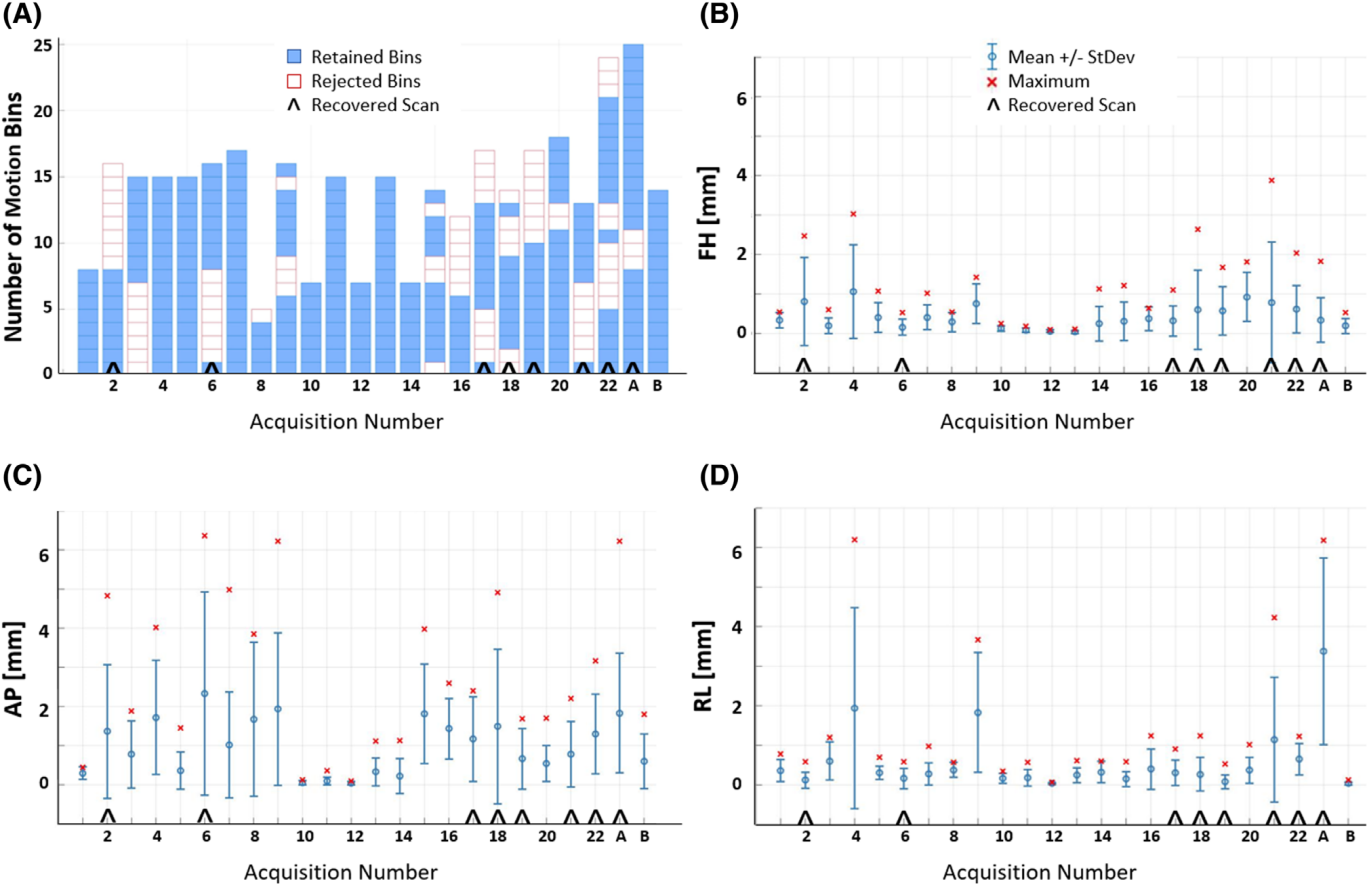
(A) A summary of the number of consecutive, 15 s motion bins that were acquired and manually retained (blue) or rejected (white) in motion correction, temporally along acquisition of 22 healthy volunteers and two aortic coarctation patients (A and B), including eight recovered scans (denoted by ^ above). Bins were excluded based on pixel shift values and vessel visibility within the angiogram. Acquisition bars that are completely blue indicate that all motion bins were retained. (B–D) Mean and SD of the absolute values of bulk motion translations in each direction (FH and AP, in‐plane; RL, through‐plane) for each subject, with maximum shifts denoted by an *x*. The reported shifts correspond to translations determined for retained motion phases. AP, anteroposterior; FH, foot–head; RL, right–left.

The range of absolute bulk motion estimations for all 24 successfully motion‐corrected acquisitions is shown in Figure 5, for translations in three orthogonal dimensions. Bulk motion phases were manually excluded in 14 of these acquisitions.

### 3.2 Visualization

**FIGURE 6:**
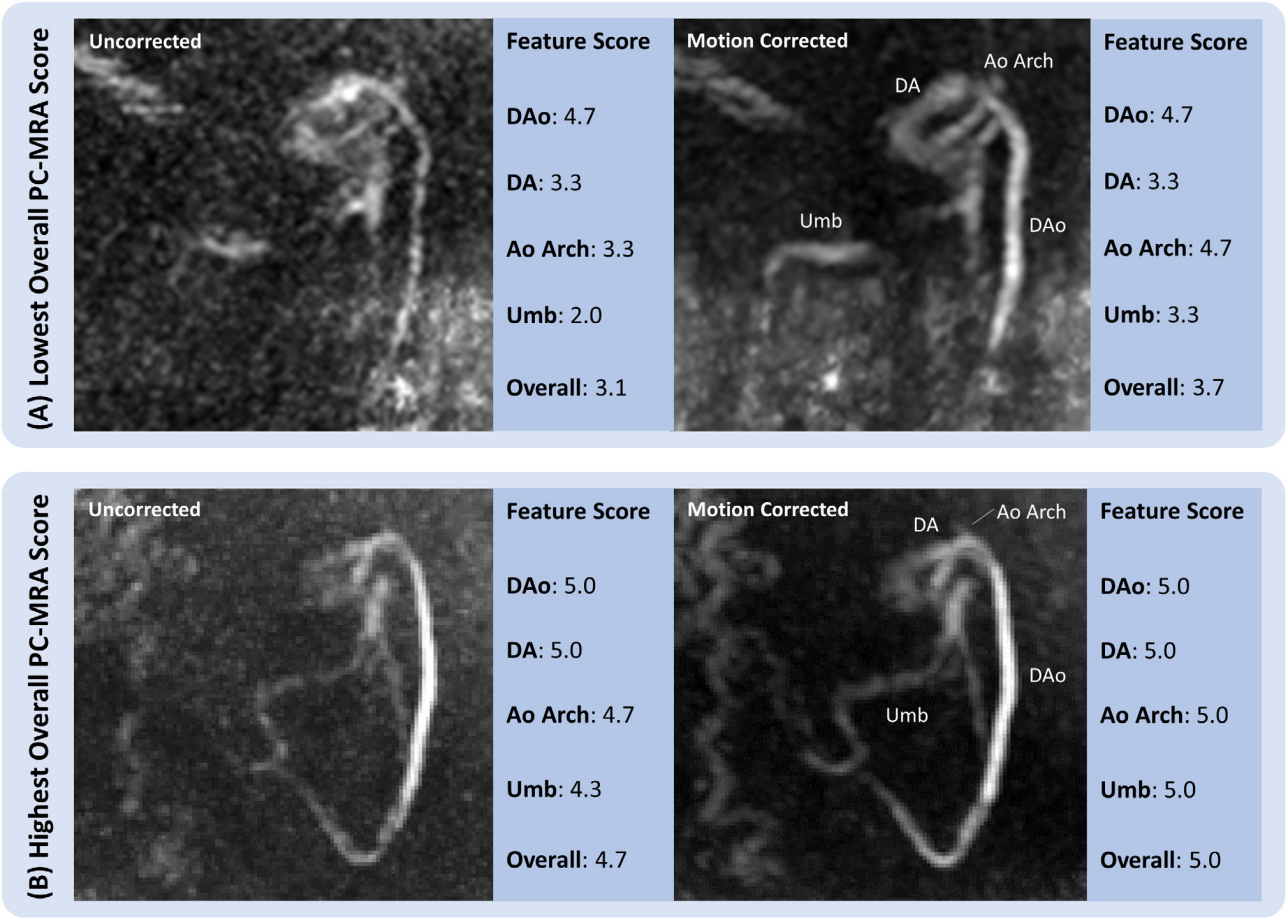
Visualization improvements in two maximum intensity projection examples (Acq. 3 and 5 in Figure 4, both with 1.25 mm acquired slice thickness), with separate feature scores for the DAo, DA, Ao Arch, Umb, and an overall averaged score. Panels depict (A) a dataset with the lowest uncorrected scores (uncorrected overall = 3.1; motion corrected = 3.6), and (B) with the highest motion‐corrected scores (uncorrected overall = 4.6; motion corrected = 4.75). The bulk of signal lost in (A) has been recovered in the DAo, and the blurring around the ductus arteriosus, and aortic arch has been reduced with motion correction. The relatively motionless fetal acquisition in (B) benefits from motion correction with improved vessel clarity and overall speckle‐noise reduction. Ao Arch, aortic arch; DA, ductus arteriosus; DAo, descending aorta; Umb, umbilical veins.

**FIGURE 7:**
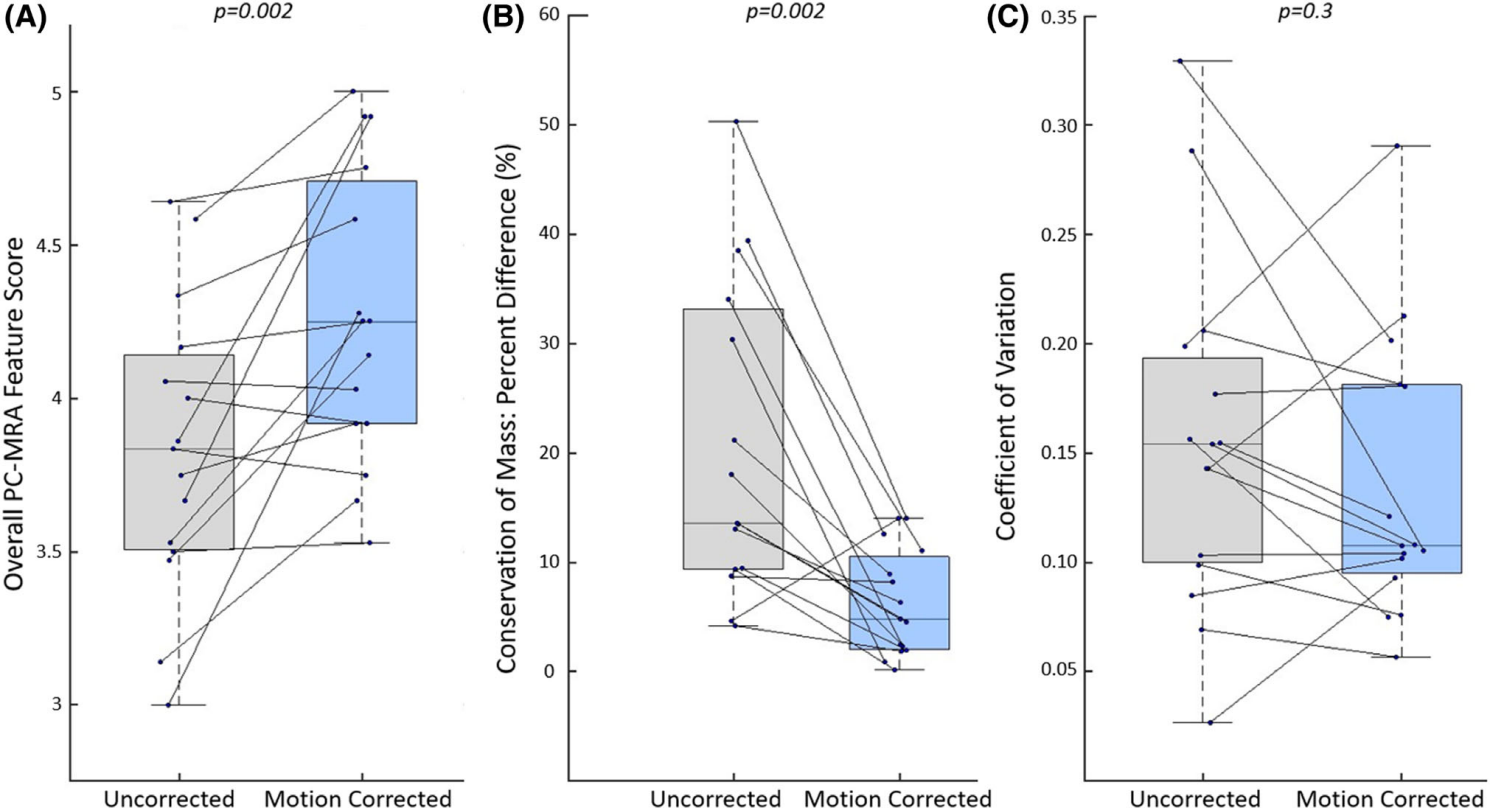
Overview of the changes in image quality and flow reliability metrics from uncorrected (gray) to motion‐corrected (blue) 4D flow in 15 healthy fetal participants. (A) Ratings of anatomical visibility in PC‐MRA images averaged across three observers. Overall scores are determined as the mean feature scores on the Likert scale of the descending aorta, ductus arteriosus, aortic arch, and umbilical veins for each uncorrected and motion‐corrected dataset. Improvements were observed in 12/15 datasets with motion correction. Uncorrected median (IQR): 3.83(0.63); motion‐corrected median(IQR) 4.25(0.79) (*p* = 0.002). (B) CoM at the insertion of the ductus arteriosus into the aorta was improved with motion correction in 14/15 4D flow datasets. CoM median (IQR) percent difference was 14% (24%) in uncorrected and 5% (9%) in motion‐corrected images (*p* = 0.002). (C) CoV along the DAo had reduced values with motion correction in 11/15 scans, median (IQR) value of 0.15 (0.09) in uncorrected images and 0.11 (0.09) in motion‐corrected images (*p* = 0.30). CoM, conservation of mass; IQR, interquartile range.

Feature scores for PC‐MRA image quality for 15 uncorrected and motion‐corrected healthy participant datasets showed significant overall visual image improvement with motion correction, as demonstrated for two examples in Figure 6. There was moderate agreement between the three observers according to the overall average ICC for interrater consistency (ICC = 0.74, 95% confidence interval = 0.54 < ICC <0.86). Feature scores were highest in the DAo, with identical median(interquartile range [IQR]) scores for uncorrected and motion‐corrected data scoring of 5(0.33) (*p = 0.3*). The remaining features had uncorrected and motion‐corrected data median(IQR) scores, respectively, as follows: aortic arch 3.67(0.92) and 4.00(0.92) (*p* = 0.03); DA 4.00(0.67), and 4.33(1.00) (*p* = 0.04); ductus venosus scores: 2.67(0.92) and 3.33(1.33) (*p* = 0.002); and overall scores: 3.83(0.63) and 4.25(0.79) (*p* = 0.002), which represents an average of the feature scores. The relative qualitative changes with motion correction for the 15 compared datasets are shown in Figure 7A.

### 3.3 Flow quantification and validation

The effect of motion correction on quantification metrics for each dataset is illustrated in Figure 7B,C. Evaluation of CoM for the DA insertion in 15 acquisition datasets indicated a significant improvement in CoM with motion correction, with lower percent difference values in 14/15 scans. Median (IQR) percent difference for CoM was 14% (24%) in uncorrected images and 5% (9%) in motion‐corrected images (*p* = 0.002). CoV along the DAo had reduced values with motion correction in 11/15 scans, median (IQR) value of 0.15 (0.09) in uncorrected images, and 0.11 (0.09) in motion‐corrected images (*p* = 0.30).

**FIGURE 8:**
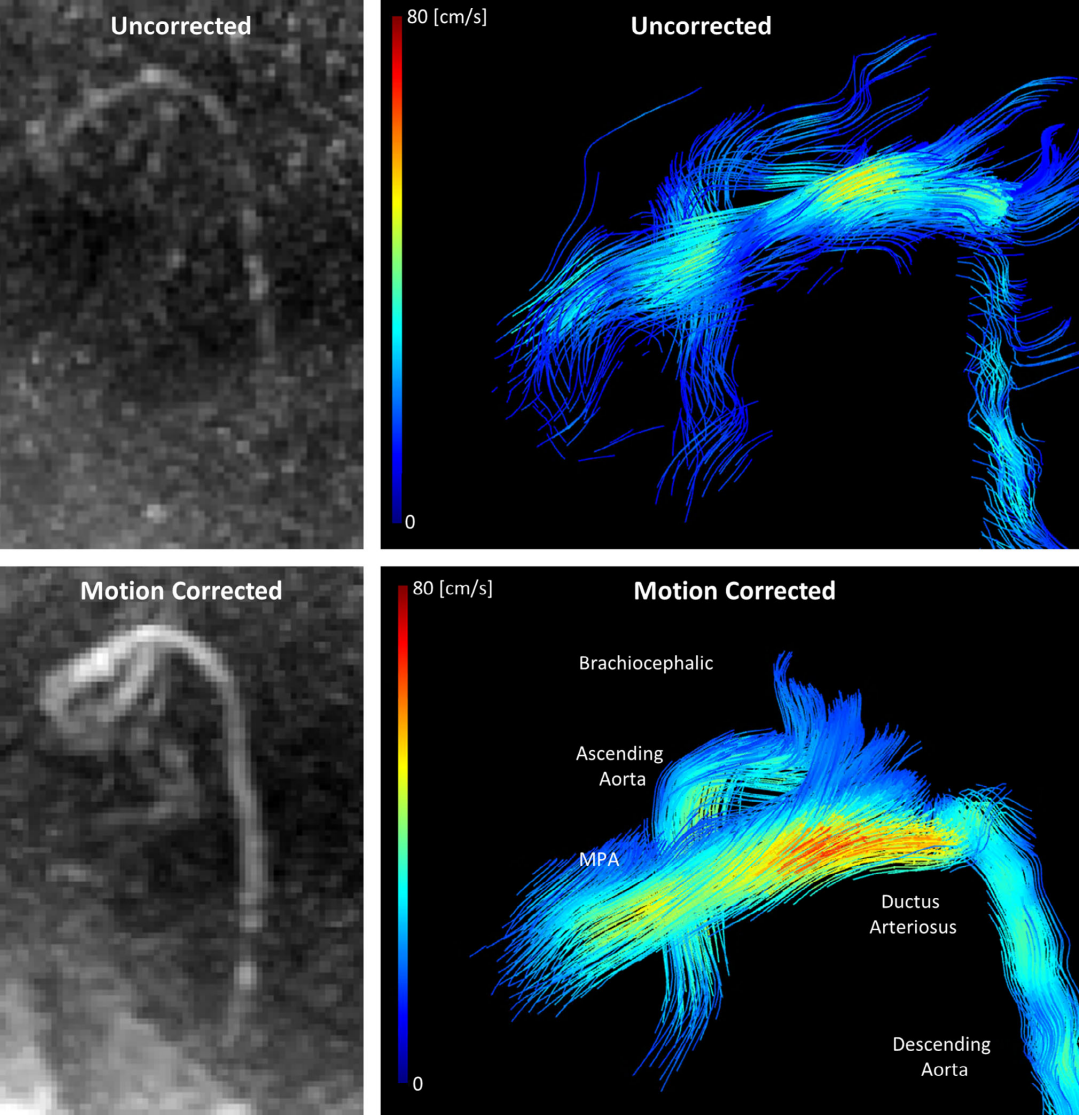
Comparison of PC‐MRA and velocity streamlines at peak systole for an unusable uncorrected scan (Acq. 19), using the segmentation of the motion‐corrected acquisition, and motion‐corrected 4D flow MRI data in a healthy participant, representing a “recovered” acquisition. MPA, main pulmonary artery.

An example of the visual impact of the motion‐correction algorithm in recovered acquisitions is given in Figure 8, showing the ductal insertion into the descending aorta of a healthy fetus. The motion‐corrected 4D flow image has improved flow streamline coherence in the DAo, DA, and aortic isthmus, with higher peak flow in the DA and improved flow through the brachiocephalic arteries.

**FIGURE 9:**
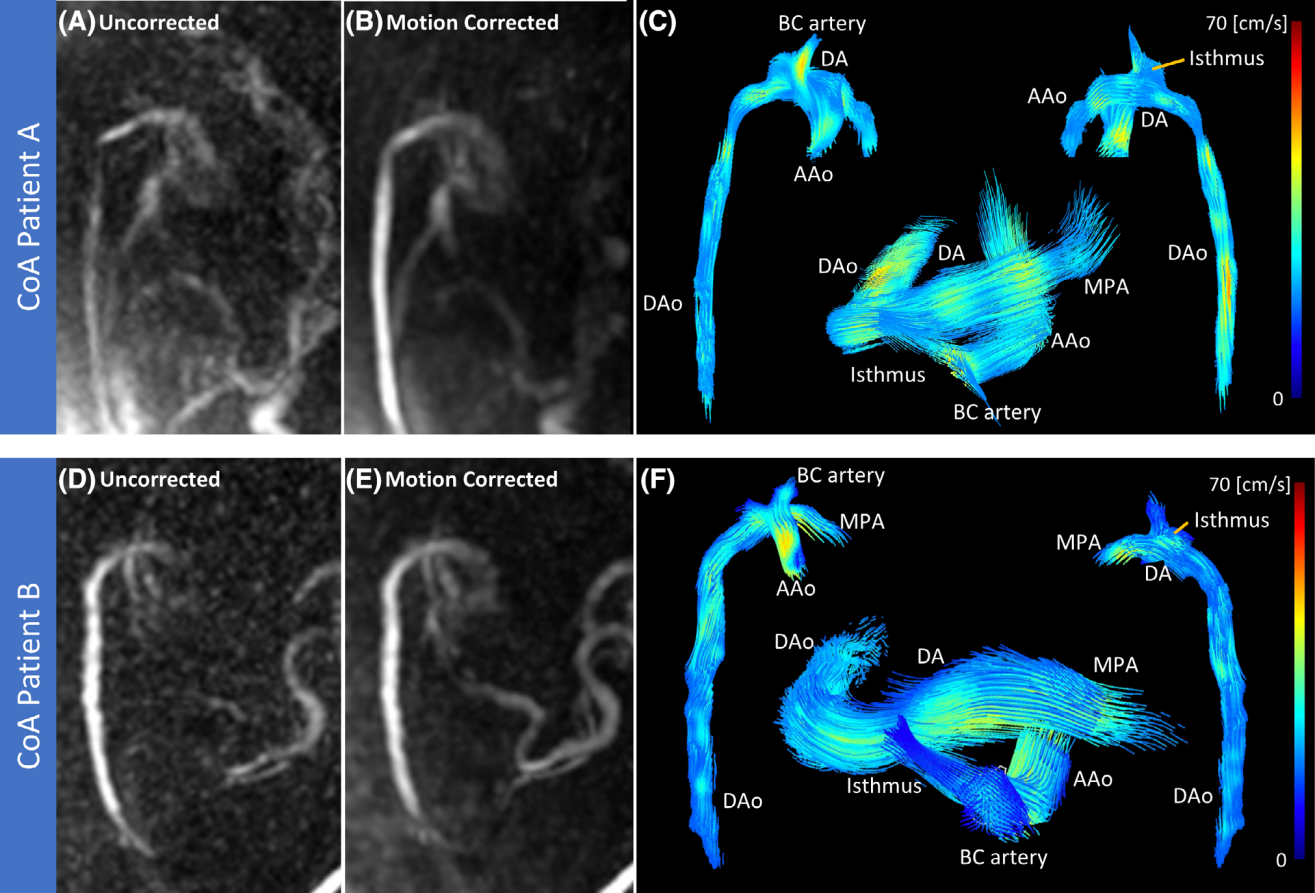
Differences with motion correction in two confirmed CoA patients, with (A, D) uncorrected and (B, E) motion‐corrected PC‐MRA maximum intensity projections, and (C, F) 4D flow images at peak systole from motion‐corrected data. The loss of signal along the descending aorta and at the aortic insertion in Patient A was recovered with motion correction, allowing for segmentation and flow evaluation. Anatomical features such as the DAo, AAo, DA, BCA, and aortic isthmus are observed in both patients. The narrowed isthmus, where the arch inserts on top of the DA, is characteristic of CoA. AAo, aortic arch; BC, brachiocephalic artery; DA, ductus arteriosus; DAo, descending aorta; MPA, main pulmonary artery.

Finally, PROUD 4D flow with motion correction was applied to two example patients with confirmed postnatal CoA to evaluate the feasibility of utilizing this technique for 4D flow in a subset of the target population for fetal CMR, shown in Figure 9. Patient A is a recovered acquisition, with unsuccessful segmentation in the uncorrected reconstruction, and therefore does not have a quantitative comparison. CoM in patient B was improved from 15% (uncorrected) to 0.0% (motion corrected); CoV was reduced from 0.15 (uncorrected) to 0.12 (motion corrected).

## 4 DISCUSSION

In this study, we propose a method for motion correction in fetal 4D flow MRI and present evidence that compensation of maternal breathing and fetal bulk motion provide qualitative and quantitative improvements to image quality and flow‐based measurements. The described approach included estimates of translations due to stochastic fetal movements and maternal respiratory gating, and the final CS reconstruction with CORe,^29^ which incorporated an additional regularization parameter for outlier rejection. Results for qualitative and quantitative flow comparison were shown for 15 healthy, third‐trimester fetal acquisitions, with significant improvements in visual image quality, greater contrast between blood vessels, and background in PC‐MRAs—and significantly more internally consistent flow quantification based on CoM measures. Most importantly, yet difficult to quantify given the lack of any uncorrected result for comparison, seven of the healthy volunteer acquisitions that were previously unusable before motion correction were recovered with the described approach, allowing anatomical segmentation and flow visualization. Two additional fetal patients with postnatally confirmed CoA were included to demonstrate the feasibility of motion‐corrected 4D flow in a clinically relevant population, including one of whom which was recovered from failure with motion correction, and the other with lower CoM and CoV after correction.

Achieving a sufficient SNR is a significant challenge in fetal flow measurements with MRI, especially when targeting low acquisition times and high spatiotemporal resolutions recommended for fetal imaging (in‐plane voxel size <1.3 mm, temporal resolution <30 ms).^8^ Fetal heart rates range from 120 to 160 beats per minute, and the diameter of major vessels is roughly 5 mm in the mid‐third trimester.^35^, ^36^ For fetal patients with CHD, such as CoA, the important task of resolving anatomical variations is often more difficult due to narrowed vessels and abnormal flow patterns. As a result, most volumetric fetal flow measurements to date have depended on signal enhancement from inflowing blood in 2D acquired slices, combined with slice‐to‐volume reconstructions techniques or accelerated imaging, with in‐plane motion correction for scans greater than 5 min.^19^, ^20^, ^37^ We demonstrate that, despite increasing relative acceleration factors, respiratory gating, and manual bulk motion phase removal, the motion‐corrected PROUD 4D flow acquisition method can provide sufficient vessel contrast for clear visualization with a single acquisition at 3 T, with a median scan time of 3.9 min. To achieve this, the approach had to balance scan time, resolution, and SNR, necessitating a reduction in resolution below the aforementioned recommended values.

Motion is inevitable in fetal CMR. Addressing this is crucial for advancing 4D flow MRI in the fetus toward clinical viability, as indicated by the high rates of scan failure due to motion demonstrated in prior work (25%)^22^ and in this study (12/27 (44%) for the uncorrected technique and 5/27 (19%) for the motion‐corrected technique). Moreover, reducing motion artifacts for effective visualization is essential for fetal CMR's diagnostic efficacy in complex CHD cases, particularly when echocardiography fails to provide a conclusive diagnosis before time of delivery. Our findings show significant visual improvements across all features of interest with the implementation of motion correction, as well as recovered information from highly corrupted scans, enhancing the performance of flow quantification beyond that of uncorrected data. The signal and flow patterns within the smaller and more tortuous fetal vessels may benefit from the outlier reduction achieved by the CORe algorithm, enhancing the clarity of the extracardiac anatomy.

Notably, CoM error with the motion‐corrected approach was markedly lower (more than three‐fold) than a recent report in 30 subjects using an uncorrected Cartesian approach, where CoM error was, on average, 19% ± 12%.^26^ Furthermore, CoM was significantly and considerably improved with motion correction in this study, bringing error levels from 14% near to that recommended in the 4D flow MRI consensus statement (<5%).^34^ In the uncorrected images, errors were sometimes severe, with flow in the DA and aortic isthmus at times becoming indiscernible, leading to reliance on segmentation for vessel localization. This issue was likely a result of fetal bulk motion throughout the scan, resulting in image blurring varying in severity, and was likely exacerbated by moving vessels throughout the fetal cardiac cycle.^8^ Whereas fetal bulk motion correction mitigated many of these issues of incoherent flow, physiological extracardiac motion from the fetal heartbeat remained in the motion‐corrected images, highlighting the importance of time‐resolved contours in analyzing flow, particularly for accurately capturing the small‐scale flows in the vessels. Although there is an improvement in measured CoV in 11 of 15 datasets, this measurement showed no significant impact of motion correction on flow precision along the DAo. Several limiting factors could be attributed to the differences in the results of these two consistency criteria, such as the nature of contour placement, which is automatic in the MatLab (MathWorks)‐based flow processing tool, the static segmentation‐based contours, and the presence of the intercostal arteries, gradually reducing flow along the length of the DAo.

In summary, this work provides evidence that the free‐running (pseudo)–random 4D flow sampling approach presented here offers advantages over conventional CS 4D flow methods for fetal CMR.^38^ A strength is the ability to recover either incomplete acquisitions due to loss of cardiac gating caused by motion, or due to motion corruption in the MRI signal. Here we focus on one aspect: the ability to recover motion‐corrupted data. Future work will extend to recovering partially acquired images where gating and thus scan failures occur due to motion. Furthermore, this framework allows for broad flexibility to perform motion correction in the reconstruction steps, and multiple approaches can be used to address the varied types of motion encountered in time‐resolved fetal CMR, whether periodic, stochastic, or occurring at small or large scales. This capability has been demonstrated in challenging settings such as adult coronary and arrhythmic flow imaging.^39^, ^40^ Additionally, the integration of CORe enables readout‐based motion suppression, which results in high‐quality imaging despite the complexities of fetal motion.

As mentioned, the approach is extendable, allowing more advanced motion‐correction techniques to be employed in future development, such as leveraging direct feedback on fetal movement during acquisition or managing complex fetal motion beyond simple translations during registration as utilized here. One limitation to the presented method is the use of temporally fixed motion bin lengths in bulk motion correction, regardless of the true motion states of the fetus. Additionally, heavily corrupted bins had to be manually excluded using visual inspection, a subjective and time‐consuming process. The signal obtained from the DUS device could provide valuable information on fetal motion states, enabling automatic data acceptance criterion and eliminate the need for manually discarding phases with excessive movement. Additionally, correcting beyond rigid translations, which currently allows for the simple application of bulk motion shifts to k‐space data without affecting velocity encoding, would account for the realistic fetal behavior that is relatively unrestricted in the womb. Advancements such as these could increase the efficacy of 4D flow exams beyond the 19% failure rate shown here and 25% in previous feasibility studies.^22^

This work has additional important limitations to consider. First, given the challenges of fetal blood flow measurements, the technique is limited to internal validation and lacks comparison to conventional methods for accelerated fetal 4D flow. Thus, an extended validation is a natural next step and ongoing. However, comparison between acquisition methods is inherently difficult because a fetus can have a wide variety of motion within a single CMR exam. Similarly, we strategically present flow ratios as opposed to absolute quantitative flow values; with the limited temporospatial resolution available, reporting accurate flow values with this method requires additional investigation. In future fetal 4D flow studies, the ability to correct for and control motion‐related artifacts could lead to improved spatial and temporal resolution compared to what is presented here.

## 5 CONCLUSION

This work demonstrated the feasibility of a 4D flow acquisition with motion correction in both healthy third‐trimester fetal participants and patients with aortic coarctation, resulting in significant visual and quantitative improvements. The approach shown here demonstrates superior image quality and flow quantification due to improved motion robustness enabled by its adaptable reconstruction capabilities, particularly with the integration of the CORe model. Further investigation into advanced motion‐correction methods, including nonrigid registration and motion‐acceptance criteria, is expected to enhance image quality, thereby minimizing motion‐related errors during prolonged scans.

## Supporting information


**Video S1.** Animated version of Figure 1: (a) PROUD data is continuously sampled with a pseudo‐spiral k‐space trajectory. (b) Data is retrospectively limited to the maternal expiratory phase, roughly 60% of data. (c) For bulk motion detection, data is binned into dynamic bins with a temporal resolution of 15 s. CS reconstruction and PC angiogram calculation are performed for all bins. Then, rigid monomodal registration of the angiogram frames corrects for fetal bulk motion over the course of data acquisition. The red blocks denote rejected bins, either for large translational shifts or for a loss of signal observed in the bin's angiogram. The obtained translations of the remaining bins were directly applied to the original k‐space data. (d) A Doppler ultrasound gating device provides temporal fetal cardiac information that guides (e) 4D flow CS reconstruction with CORe. 4D flow, depicted by the magnitude (*M*) and three orthogonal velocity (*V*_x_, *V*_y_, *V*_z_) images at peak systole, was reconstructed.

**Table S1.** Description of each participating mother and fetus (15 volunteers and 2 confirmed aortic coarctation patients) and respective acquisition(s). Fetal gestational age (GA) ranged from 32 to 36 weeks. Scan numbers correspond to those attributed in Figure 5, where a description of applied motion correction for each acquisition is given. The final column addresses the amount of k‐space data per cardiac bin utilized in the final reconstruction after motion correction, relative to full k‐space sampling.
